# Kinetics of Fumonisins B_1_ and B_2_, Deoxynivalenol, and Deoxynivalenol-3-β‑d‑glucoside during Baking of Wheat–Maize Bread

**DOI:** 10.1021/acs.jafc.5c02400

**Published:** 2025-09-05

**Authors:** Alexandre Vicens-Sans, Sonia Marín, Vicente Sanchis, Antonio J. Ramos, Francisco Molino

**Affiliations:** Applied Mycology Unit, Food Technology, Engineering and Science Department, 16739 University of Lleida, AGROTECNIO-CERCA, Av. Rovira Roure 191, 25198 Lleida, Spain

**Keywords:** deoxynivalenol, deoxynivalenol-3-β-d-glucoside, fumonisin B_1_, fumonisin B_2_, bread, baking, kinetics

## Abstract

Deoxynivalenol (DON), B-type fumonisins (FBs), and deoxynivalenol-3-β-d-glucoside (DON-3-Glu) are mycotoxins synthesized by *Fusarium* species that infect wheat and maize. Mycotoxins pose a food safety problem due to their toxicity. This work studied the evolution of DON, FBs, and DON-3-Glu levels, alongside their kinetics, to develop predictive tools for their fate during baking. Maize–wheat (40–60%) flour was contaminated with 1433.17 ± 43.40 μg DON/kg and 1383.52 ± 31.44 μg FBs/kg. Bread loaves were baked from 160 to 220 °C, over seven time periods. Samples were analyzed by HPLC. At the end of baking, DON decreased by 21–33%, FB_1_ by 45–66%, and FB_2_ by 33–53%. By contrast, DON-3-Glu increased by 201–696%. Baking may be enough to meet maximum legal limits for FBs but may not ensure safe DON levels. All mycotoxin variations followed first-order kinetics. The derived parameters can be used to predict their fate under various conditions, ensuring food safety and product quality.

## Graphical abstract

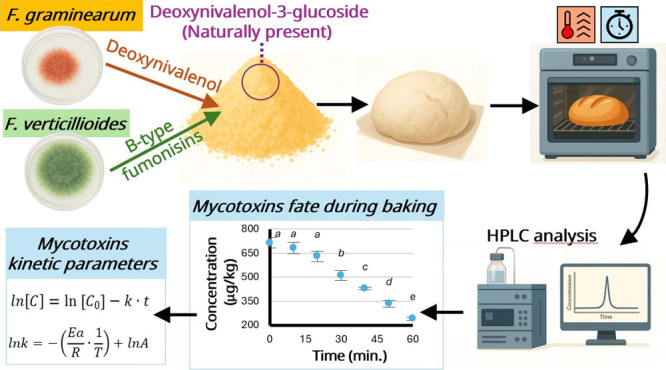

## 1 Introduction

Mycotoxins are secondary metabolites biosynthesized by specific mold genera, classified as mycotoxigenic. These compounds are biosynthesized during the secondary metabolism once molds have infected and developed in plants. Mold infections can occur either during the pre- and postharvest of crops.^1^ *Aspergillus*, *Penicillium*, and *Fusarium* genera are primary producers of mycotoxins, many of which are currently regulated under EU Regulation 2024/1022. Wheat and maize are cereals that are frequently infected with *Fusarium* spp. These cereals represent over half of the worldwide total cereal production. *Fusarium* species produce different kinds of mycotoxins, depending on the cereal. When wheat is infected, deoxynivalenol (DON) is mainly synthesized, whereas maize infection leads to the production of fumonisins (FUM) and DON, among other toxins.^2–4^

FUM, with its 28 different analogues, is a very diverse group. However, the occurrence of B-type fumonisins (FBs) surpasses the other types.^5^ In nature, fumonisin B_1_ (FB_1_) represents 70% of the FB group, followed by fumonisin B_2_ (FB_2_), with an occurrence ranging from 15 to 25%.^6^

DON belongs to the trichothecene B group and has the greatest occurrence among all trichothecenes.^7^ Deoxynivalenol-3-β-d-glucoside (DON-3-Glu) is classified as a biologically modified form of DON,^8^ meaning that it is formed because of the hosting plant defense mechanism, which, via glycosylation, inactivates DON as part of the detoxification process. DON-3-Glu is less harmful than its parent form; however, it can revert to DON through hydrolysis during fermentation or digestion.^9^

Recent studies on the influence of climate change in *Fusarium* spp. indicate that their populations may increase to dangerous levels and spread to new geographic latitudes over the years.^10,11^ Additionally, due to climate change, the presence and distribution of their mycotoxins are changing in cereals.^12^ Also, Cendoya et al.,^13^ focusing on the occurrence of FBs in wheat and maize, indicated that, between 2008 and 2018, there was an increase in FB occurrence in maize and wheat. These data suggest that over the years, wheat and maize have become concerning sources of either DON or FBs in several cereal-based products.

Wheat and maize are the dietary basis for many countries and cultures around the world. Bread stands as one of the main cereal-based products produced worldwide. During its making process, baking has been reported to influence mycotoxin levels in different ways. In the literature, there are studies where DON levels were significantly reduced,^14,15^ as well as others where the final levels were higher than the initial ones.^16,17^ For DON-3-Glu, a similar scenario appears: some studies conclude that mild baking temperatures seem to induce its liberation from the matrix, leading to increased levels,^18,19^ while others observe the opposite scenario.^16,20^ Regarding FBs, data published show a reduction when exposed to different baking conditions.^21,22^ The high heat resistance of mycotoxins, combined with processing parameters such as baking time, product mass, and matrix type, may influence their fate during processing.

The aim of this study is to investigate the fate of DON, DON-3-Glu, FB_1_, and FB_2_ under various baking conditions to determine which conditions are most effective in ensuring optimal mycotoxin degradation while maintaining the physicochemical characteristics of the product. For this, kinetic parameters of the degradation/liberation reactions were estimated.

## 2 Materials and Methods

### 2.1 Chemicals and Reagents

DON, DON-3-Glu, and FB_1_ and FB_2_ commercial standards were acquired from Sigma-Aldrich (Saint Louis, USA). Methanol and acetonitrile of HPLC grade (≥99.98% purity) were sourced from Thermo Fisher Scientific (Waltham, USA). Ultrapurified water was produced by a Milli-Q SP system (Millipore Corp., Brussels, Belgium). A 0.10% acetic acid solution with glacial acetic acid (VWR Chemicals, Llinars del Vallés, Spain) and Milli-Q water was prepared. PBS solution was made by dissolving 0.20 g of potassium chloride, 0.20 g of potassium dihydrogen phosphate, 1.16 g of anhydrous disodium phosphate, and 8.0 g of sodium chloride in 1 L of distilled water. The pH of the PBS solution was then adjusted to 7.4 using 1 M hydrochloric acid. PBS reagents were obtained from Panreac (Castellar del Vallès, Spain), and only sodium chloride was acquired from Thermo Fisher Scientific (Waltham, USA). OPA solution was prepared by dissolving 40 μg of *o*-phthaldialdehyde (Sigma-Aldrich, Saint Louis, USA) in 1 mL of methanol, 10 mL of sodium tetraborate (Sigma-Aldrich, Saint Louis, USA), and 50 μL of molecular biology grade mercaptoethanol (Scharlab, Barcelona, Spain). Immunoaffinity chromatography columns (IAC) DONPREP were used for DON and DON-3-Glu cleanup and IAC FUMONIPREP for FB_1_ and FB_2_ cleanup; both were purchased from R-Biopharm (Darmstadt, Germany).

### 2.2 Flour Contamination

Commercial noncontaminated maize flour (Farinera La Segarra S.A., Maldà, Spain) was contaminated in the lab with *Fusarium* molds. The strains used were a *F. graminearum* DON producer (strain F.45) and a *F. verticillioides* FB producer (strain F.109), from the collection of the Department of Food Technology, Engineering and Science at the University of Lleida (Spain). Before, those strains were cultured in Potato Dextrose Agar (PDA) medium and incubated for 7 days at 25 °C. In parallel, 3 kg of maize flour and 1 L of distilled water were autoclaved at 121 °C for 15 min. Afterward, 20 g of sterile maize flour and 1 mL of distilled water were placed in Petri dishes. Half of the Petri dishes were inoculated with agar plugs of *F. graminearum* (F.45) and the other half with *F. verticillioides* (F.109) and incubated for 20 days at 30 °C. This incubation was performed by separating the Petri dishes into groups of 12 and placing them inside different airtight containers, each one with 2 flasks filled with 200 mL of sterile water, to maintain a high humidity environment. Lastly, both DON- and FB-contaminated flours were dried separately at 40 °C for 48 h in a UF 160TS Memmert oven dryer (Memmert GmbH + Co.KG, Schwabach, Germany).

### 2.3 Bread Making and Experimental Setup

Each loaf was prepared by mixing 50 g of commercial strong wheat flour (Harinera La Meta S.A., Lleida, Spain), 33 g of contaminated maize flour, 54 mL of water, 1 g of compressed yeast (Lesaffre Ibérica, Valladolid, Spain), and 1.7 g of salt. The 60/40 wheat/maize ratio was chosen as it is commonly used and gave a suitable texture to the bread. Compressed yeast was previously dissolved in warm water. The mixture was kneaded for 10 min. All prepared doughs were weighed 100 g before baking. Four different temperature levels were selected with different baking times. At 160 °C, loaves were baked up to 90 min; at 180 °C, up to 75 min; at 200 °C, up to 60 min; and at 220 °C, up to 40 min. In all cases, 7 sampling times were set throughout the process. All experiments were performed in triplicate, which resulted in 84 loaves. All loaves were dried in the Memmert dryer at 40 °C for 24 h and stored under refrigeration (5 °C). Samples were weighed before and after baking and after drying.

### 2.4 Standard Preparation

Stock standard solutions had initial mycotoxin concentrations of 872 μg/mL DON, 50 μg/mL DON-3-Glu, 250 μg/mL FB_1_, and 500 μg/mL FB_2_. DON and FB_1_ calibration curves were prepared at 2.0, 1.5, 1.0, 0.75, 0.50, and 0.10 μg/mL. FB_2_ calibration curves contained half of the values of FB_1_. DON-3-Glu calibration curves were made with 0.50, 0.20, 0.10, 0.05, and 0.02 μg/mL. All R-squared values of the calibration curves were above 0.99. Methanol was used as the solvent either to resuspend the commercial standards or to prepare the calibration curves. All standards were stored at −18 °C until their use. DON was the only one standard, whose concentration could be confirmed by a UV spectrophotometer (UV-1600PC; VWR, Radnor, USA), according to the 49th chapter of AOAC Official Methods of Analysis,^23^ at 219 nm and with an extinction coefficient of 7040.

### 2.5 Mycotoxin Extraction, Cleanup, and Detection

#### 2.5.1 DON and DON-3-Glu

Samples were ground with a batch mill IKA A11 basic (IKA-Werke, Staufen, Germany). Afterward, 5 g was weighed for DON and DON-3-Glu extraction. Then, they were mixed with 40 mL of ultrapurified water and stirred magnetically for 15 min. Subsequently, the samples were centrifuged at 9000 rpm and 4 °C for 10 min. The resulting supernatants were filtered with Whatman glass microfiber filters (Cytiva, Barcelona, Spain) and vortexed for 1 min. Later, 8 mL of each sample was loaded to a DONPREP IAC column for cleanup. Next, columns were washed with 10 mL of ultrapurified water. DON and DON-3-Glu were eluted with 1.5 mL of methanol. To ensure an optimal recovery, methanol was backflushed 3 times. Finally, an additional 1.5 mL of methanol was loaded. All samples were dried under a nitrogen stream at 40 °C.

Once dried, samples were resuspended with 1 mL of the mobile phase. This solution was made by mixing ultrapurified water, methanol, and acetonitrile (90:5:5 v/v/v). Samples were then homogenized by vortex mixing and filtered through 0.22 μm pore size PTFE filters (Dominique Dutscher SAS, Bernolsheim, France). Mycotoxin detection was performed by Agilent 1260 Infinity II High-Performance Liquid Chromatography equipment coupled with an Agilent 1260 Diode Array Detector HS (HPLC-DAD). DON and DON-3-Glu were separated from the rest of the sample by a Gemini C18 column (150 × 4.6 mm with 5 μm particle size and 110 Å pore size; Phenomenex, Torrance, USA), which served as the stationary phase and warmed-up at 40 °C. 50 μL of each sample was injected into the HPLC system at a 1 mL/min mobile phase flow rate. DON and DON-3-Glu were detected at 220 nm.

#### 2.5.2 FB_1_ and FB_2_

Samples were ground with a batch mill, and 5 g was weighed for FB_1_ and FB_2_ extraction. Then, they were mixed with 25 mL of ultrapurified water, methanol, and acetonitrile (50:25:25 v/v/v) and stirred magnetically for 15 min. Later, samples were centrifuged at 9000 rpm and 23 °C for 10 min, filtered with Whatman glass microfiber filters, and vortexed for 1 min. Then, 10 mL was mixed with 40 mL of PBS. All mix was loaded in a FUMONIPREP IAC for cleanup. Columns were washed with 20 mL of PBS. By loading 1.5 mL of methanol, FBs were eluted, using backflushing 3 times. An additional 1.5 mL of methanol was loaded for final elution. Samples were dried under a nitrogen stream at 40 °C.

Samples were resuspended with 1 mL of ultrapurified water and methanol (50:50 v/v). Then, they were homogenized by vortex mixing and filtered with PTFE filters. FB detection was carried out by HPLC with the Agilent 1620 Fluorescence Detector Spectra (HPLC-FLD). FBs were separated from the rest of the sample by a Kinetex PFP column (150 × 4.6 mm with 5 μm particle size and 100 Å pore size; Phenomenex, Torrance, USA), which served as the stationary phase and warmed-up at 40 °C. The mobile phase components used during analysis were acetonitrile (A), methanol (M), and acetic acid 0.10% (AC). Mobile phase (A-M-AC%) was pumped in gradient mode, with the following conditions: 0–10 min (15–0–85%); 10–14 min (5–61–34%); 14–16 min (5–72–23%); and 16–20 min (15–0–85%). Before the injection, 15 μL of every sample was automatically derivatized with 35 μL of the OPA reagent and mixed for 30 s. Mobile phase was pumped at a 1.2 mL/min flow rate. FBs were detected at 335 nm excitation and 440 nm emission wavelengths.

### 2.6 Method Validation and Performance

To study the method accuracy, for each studied mycotoxin, 11 noncontaminated bread samples were prepared (Table 1). After being ground, 7 g was taken from each sample and spiked at three different toxin levels (DON: 220, 540, and 1100 μg/kg; DON-3-Glu: 100, 250, and 500 μg/kg; FB_1_: 220, 430, and 1100 μg/kg; FB_2_: 110, 220, and 540 μg/kg). After spiking, samples were vortexed for 1 min and let stand for 2 h. Two extra samples were not spiked and were used as blanks. Mycotoxin extraction and analysis were performed, as explained in Section 2.6. Recovery rates were calculated. The limit of detection (LOD) was assessed as 3 times the signal of noise. The limit of quantification (LOQ) was calculated as 3 times of LOD.

**Table 1:** DON, DON-3-Glu, FB_1_, and FB_2_ Methods of Analysis Validation^a^

	LOD	LOQ	spiking		replicates	recovery
	(μg/kg)	(μg/kg)	level	(μg/kg)	*n*	(% ± SD (%))
DON			low	220	3	73.4 ± 3.9
	60	180	medium	540	5	85.9 ± 1.5
			high	1100	3	78.0 ± 0.41
DON-3-Glu			low	10	3	80.0 ± 8.6
	7	21	medium	25	5	79.7 ± 4.8
			high	500	3	70.7 ± 11.2
FB_1_			low	220	3	84.3 ± 12.5
	20	60	medium	430	5	97.6 ± 1.7
			high	1100	3	92.3 ± 0.76
FB_2_			low	110	3	76.7 ± 13.9
	60	180	medium	220	5	84.8 ± 3.7
			high	540	3	65.9 ± 1.2

a LOD, limit of detection; LOQ, limit of quantification; *n*, number of replicates; SD, standard deviation.

### 2.7 Statistics

RStudio (version 4.3.0) with R Commander library (version 2.8–0) was used to perform the analysis of variance (ANOVA) of the effects of temperature and time on mycotoxin levels, and the Tukey HSD test at a 95% confidence level (*p* value >0.05) was used for comparison of means. The regression analyses were carried out with Excel (version 2410).

### 2.8 Kinetic Parameter Estimation

Mycotoxin concentrations during bread baking may follow different degradation kinetic models. In this study, kinetic order equations used were zero (eq 1), first (eq 2), and second order (eq 3).

$$[C]=[C_{0}]-k\cdot t$$ 1

$$\ln[C]=\ln[C_{0}]-k\cdot t$$ 2

$$\frac{1}{\left[C\right]}=\frac{1}{\left[C_{0}\right]}+k\cdot t$$ 3

where *C* is the compound concentration at a certain time (μg/kg); *C*_0_ is the initial compound concentration (μg/kg); *k* is the degradation rate constant (min^–1^); and *t* is the process time (min).

*C* expressions of the equations were plotted against time to calculate their determination coefficients (*r* ^2^). With the *r* ^2^ comparison, the best fitting kinetic order was determined for each mycotoxin.

Both k and half-life (*t*_1/2_) were estimated from the chosen kinetic equation of each mycotoxin. For *t*_1/2_, *C* was substituted by 0.5*C*_0_ to determine the time necessary to reduce the toxin concentration to half.

To calculate the activation energy (*E*_a_), first, the logarithm of *k* (ln *k*) was plotted against the inverse of temperature (1/*T*) to obtain the slope of the resulting regression line. Then, the Arrhenius eq 4 was used to estimate each *E*_a_:

$$\ln k=-\left(\frac{E_{a}}{R}\cdot \frac{1}{T}\right)+\ln A$$ 4

where *k* is the degradation rate constant (min^–1^), *E*_a_ is the energy of activation (kJ/mol), *R* is the gas constant 0.00831 (kJ/mol K), *T* is the temperature (K), and *A* is the frequency factor.

## 3 Results and Discussion

### 3.1 Preliminary Experimental Steps

#### 3.1.1 Validation of the Method of Analysis

In general, mycotoxin recoveries (Table 1) obtained in the present study fit within the range established by the Regulation (EU) 2023/2782.^24^ The extraction method used in this study is suitable for determining DON and FB_1_ levels in the samples, while recovery is lower for FB_2_ and for DON-3-Glu at the highest spiking level.

#### 3.1.2 Flour Contamination

Commercial strong wheat flour (Harinera La Meta S.A., Lleida, Spain), along with commercial and both DON- and FB-contaminated maize flours, was analyzed by HPLC (see Section 2.5) to determine their contamination levels of DON and FBs (Table 2). To obtain a batch of maize flour to conduct the experiments, the three maize flours were mixed at specific proportions. Those quantities were calculated by applying the following system in eq 5:

$$\begin{array}{c} M\cdot C_{\mathrm{DON}}=AX+BY+CZ\\ M\cdot C_{\mathrm{FB}}=DX+EY+FZ\\ M=X+Y+Z \end{array}\}$$ 5

where *M* is the objective flour quantity (kg), *C*_DON_||*C*_FB_ is the objective concentration levels of DON||FBs (μg/kg), *A*||*D* is the reported DON||FBs levels in commercial maize flour (μg/kg), *B*||*E* is the reported DON||FBs levels in DON-contaminated flour (μg/kg), *C*||*F* is the reported DON||FBs levels in FB-contaminated flour (μg/kg), and *X*||*Y*||*Z* is the amount of each flour needed in the mix (kg).

**Table 2:** DON and FB Levels in Maize Flours

	commercial maize flour		DON-contaminated flour		FB-contaminated flour	
	(μg/kg)	eq 5	(μg/kg)	eq 5	(μg/kg)	eq 5
DON	266.35	*A*	40,763.28	*B*	260.59	*C*
FBs	491.12	*D*	161.29	*E*	40,000.00	*F*

To obtain 3 kg of maize flour (M) contaminated with 1200 μg/kg DON (*C*_DON_) and 1000 μg/kg FBs (*C*_FB_), with the concentrations of the three maize flours (Table 2), *X*-*Y*-*Z* variables from eq 5 were calculated to be 69.4 g of DON-contaminated flour, 39.2 g of FB-contaminated flour, and 2891.4 g of the commercial flour. Final concentrations once analyzed were 1433.17 ± 43.40 μg/kg for DON, 1140.01 ± 16.30 μg/kg for FB_1_, and 243.52 ± 12.01 μg/kg for FB_2_.

DON-3-Glu, a glycosylated form of DON, is naturally produced by the plant as a defense response during the *Fusarium* spp. growth. Since this metabolic process only occurs in the living plant, inoculating the flour with the mold cannot lead to the DON-3-Glu formation. Therefore, only the naturally occurring levels (7.50 ± 1.33 μg/kg) of DON-3-Glu were considered for the experiment in the prepared doughs.

### 3.2 Effects of Bread Baking

#### 3.2.1 DON and DON-3-Glu

The evolution of DON and DON-3-Glu during the baking process is represented in Figure 1.

**Figure 1:**
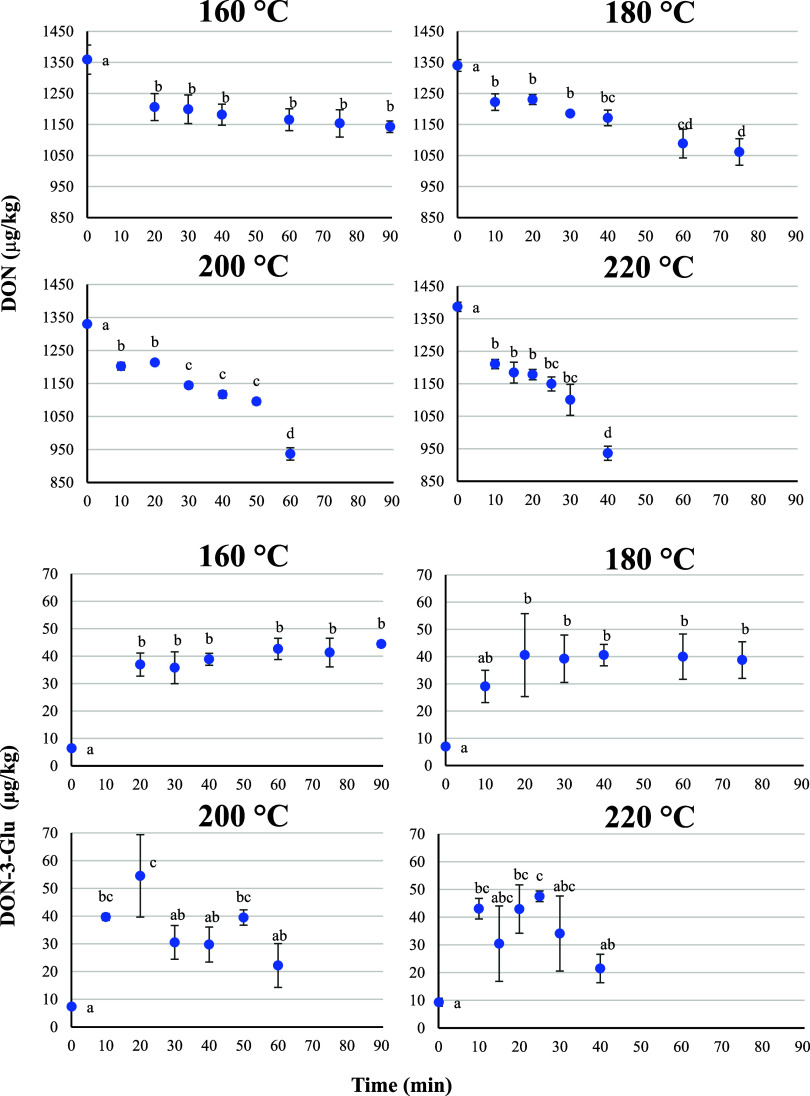
Effect of time/temperature treatments on DON and DON-3-Glu levels in bread. Error bars represent the SD of toxin concentration (*n* = 3). Different letters mean significant differences among bread samples according to the Tukey test (*p* < 0.05).

Regarding DON, there is a decrease at all the tested temperatures; the higher the temperature applied, the higher the reduction observed. At 160 °C, a statistically significant DON decrease is not observed until 60 min, with a final reduction of 15.9% ± 1.6%. At 180, 200, and 220 °C, significant reductions are observed just after 10 min, with final reductions of 20.8% ± 4.0%, 29.6% ± 2.0%, and 32.5% ± 2.3%, respectively.

Vidal et al.^15^ baked wheat-based bread (260 g) at five different temperature levels (170 to 210 °C) for four different times (45, 75, 105, and 135 min). Despite using similar baking conditions, their loaves showed higher reduction rates compared to ours. They suggested that time had a greater impact on DON levels than temperature. In the same way, Stadler et al.^25^ baked wheat bread (400 g) at three times and temperatures (15, 22, and 29 min and 185, 205, and 225 °C). They also observed that the baking time had a higher effect. Relating to the present study, ANOVA also indicated that time had a greater impact than temperature applied on DON reduction.

On the other hand, Numanoglu et al.^14^ tested DON degradation on a maize matrix by preparing traditional Turkish maize bread (20 g), baking it from 150 to 250 °C at different times (5 to 180 min). At 200 and 250 °C, DON reductions were lower than in our case: 35.7% after 30 min and 31.8% after 15 min of baking, respectively. In the same experiment, the authors compared these results with those obtained by baking larger maize bread loaves (166 g) at 250 °C for 75 min. In this case, DON reduction was significantly lower, with an 11.6% decrease in the crust and no decrease in the crumb. Previously, Numanoglu et al.^26^ baked bigger Turkish bread (1500 g) at 210 °C for 60 min. No statistical difference was found in DON levels in either the crust or the crumb compared to the initial levels. Consequently, with larger loaf sizes, smaller DON reductions were observed.

Results of the aforementioned studies suggest that maize bread presents less DON reduction at similar baking conditions and loaf sizes^14,26^ than wheat bread.^15,27^ Data obtained in the present work fall between those scenarios, showing greater DON reduction than maize bread but less than wheat bread. This can be related to the properties of those cereals. Wheat, due to the presence of gluten, forms an elastic and thin crust, allowing for greater heat transfer. In contrast, maize doughs form a denser and more rigid crust, probably leading to poorer and slower heat transfer. In relation to this, Grassi de Alcântara et al.^28^ evaluated different dough formulations. Partial wheat substitution with maize flour reduced heat transfer to the crumb (21%) and water loss (5%), compared with the control wheat doughs. They concluded that the maize composition delays heat and mass transfer during baking.

DON-3-Glu, on the other hand, reacted differently with the heat treatments. Under all temperature levels tested, DON-3-Glu showed a significant increase during the initial phase of baking (0 to 20 min), with rises of 578.9% ± 11.4% (at 160 °C), 585.9% ± 37.6% (at 180 °C), 739.8% ± 27.3% (at 200 °C), and 462.4% ± 8.6% (at 220 °C). At 160 and 180 °C, after the first 20 min of baking, DON-3-Glu levels remained stable throughout the process. However, at 200 and 220 °C, DON-3-Glu levels declined as baking continued.

Khaneghah et al.^20^ performed a meta-analysis study on the fate of DON conjugates during the elaboration of different cereal products. When baking was analyzed, among 114 data reports, 34.3% showed an increase in DON-3-Glu levels. Vidal et al.^18^ and Kostelanska et al.,^19^ authors included in the meta-analysis, concluded that the DON-3-Glu increase is not linked to DON glycosylation. Instead, it may be due to the breakage of the bonds between DON-3-Glu and the polysaccharide matrix.

Vidal et al.^29^ prepared wheat bread analogues (9 g) at four temperatures (140, 160, 180, and 200 °C) over eight times (from 5 to 40 min). Their samples showed releases between 30 and 642%. After those increases, at 180 and 200 °C, DON-3-Glu levels were rapidly reduced to <LOD levels. Meanwhile, at lower baking temperatures, the released DON-3-Glu levels remained stable throughout the treatment. DON-3-Glu decreases were not observed until 200 and 220 °C were applied for 30 min. The fact that longer time and higher temperatures were needed to reduce the increased DON-3-Glu levels may be related to the maize dough properties (aforementioned in this subsection).

DON-3-Glu release from the matrix may be linked to enzymatic activity (either from flour or microorganisms), where hydrolysis of the glycosidic bonds between DON-3-Glu and polysaccharides occurs.^15,19,30,31^ During baking, the crumb temperature increases slower than the crust, reaching a maximum temperature of approximately 100 °C.^32,33^ This slow increase in temperature during early baking allows enzymes to remain active, releasing DON-3-Glu until inactivation temperatures are reached. During later baking stages, higher temperatures break glycosidic bonds, converting DON-3-Glu to DON or other degradation products.^19^ At lower temperatures, released DON-3-Glu persists until the end of the process.

#### 3.2.2 FB_1_ and FB_2_

The evolution of both FB_1_ and FB_2_ during the baking process is represented in Figure 2. FB_1_ showed final reductions of 44.8% ± 5.1% (160 °C), 57.0% ± 4.0% (180 °C), 66.4% ± 3.9% (200 °C), and 65.1% ± 3.5% (220 °C). FB_2_ showed final reductions of 32.8% ± 15.5% (160 °C), 42.1% ± 2.5% (180 °C), 47.3% ± 1.8% (200 °C), and 52.7% ± 2.8% (220 °C). Overall, FB_2_ was less reduced than FB_1_ under all baking conditions.

**Figure 2:**
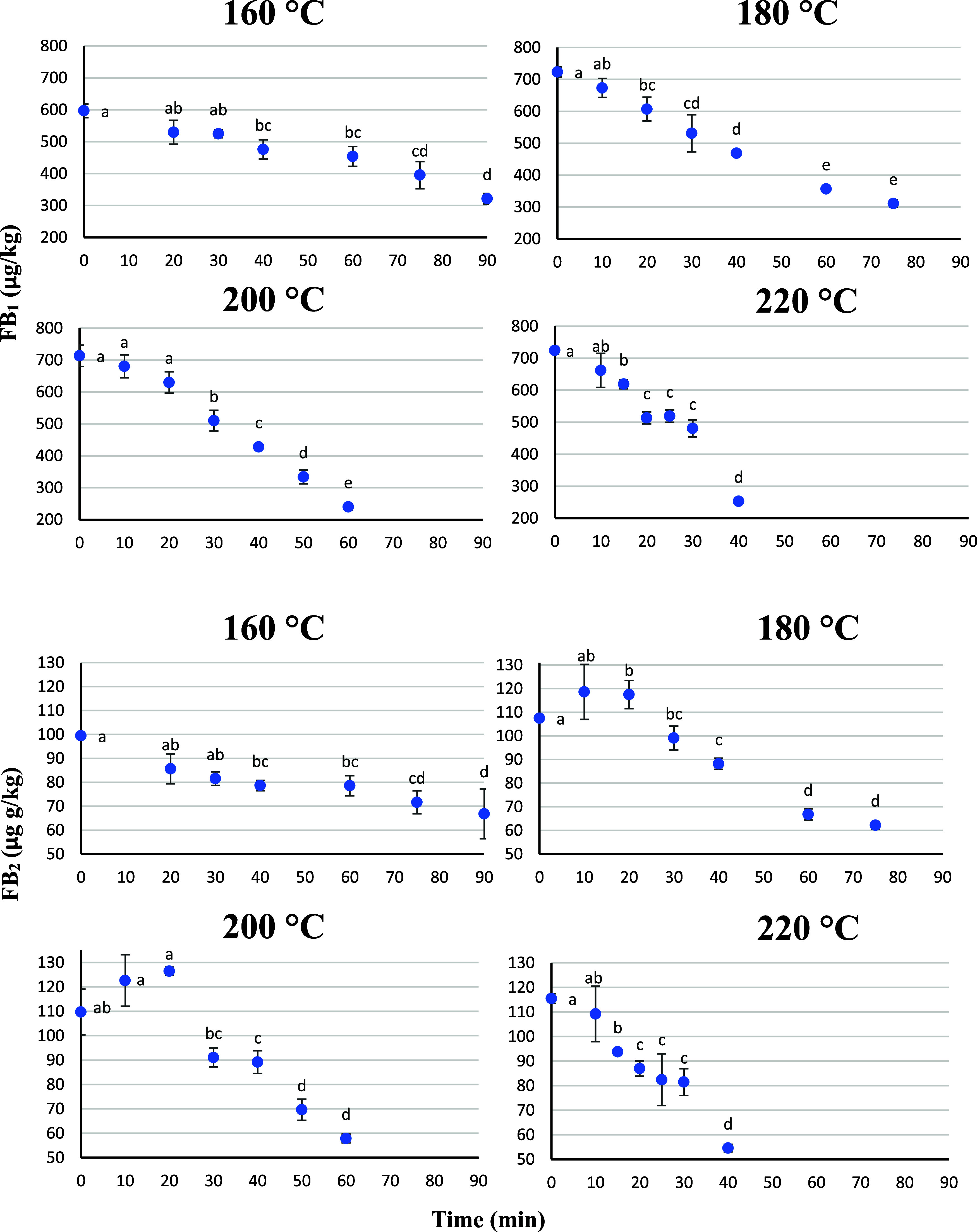
Effect of time/temperature treatments in FB_1_ and FB_2_ levels in bread. Error bars represent the SD of toxin concentration (*n* = 3). Different letters mean significant differences among bread samples according to the Tukey test (*p* < 0.05).

In the present study, until 20 min of baking, nonsignificant changes in FBs were observed. This initial apparent stability might be attributed to heat-induced transformation reactions between FBs and hidden FBs (HFBs), occurring in both directions.^22^ HFBs are FBs covalently or noncovalently bound to matrix macroconstituents. However, at higher times/temperatures, FB levels decrease as their transformations may be promoted into other derivative compounds, such as browning reaction products (BRPs), rather than solely interconversions with HFBs. The BRPs are formed when FBs are exposed to high temperature, triggering Maillard reactions in which the C2 amino group of FBs interacts with the matrix reducing sugars, forming Schiff bases. These intermediate molecules are formed and then undergo further structural rearrangements, leading to different BRPs.^22,34^

Bryla et al.^22^ baked maize bread loaves (100–150 g) for 38 min at 260 °C (lowered to 210 °C). After baking, FB_1_ and FB_2_ levels decreased by 24.1 and 40.0%, respectively. Considering HFBs, FB_1_ levels doubled initially but reduced similarly after baking. FB_2_ levels remained stable. FB_1_ to form thermally stable bonds with macromolecules explains this behavior. FB_2_ lacks the −OH group necessary to be bound. Overall, total FB and HFB reductions were 30 and 19%, respectively. Also, the HFB-FB ratio rose from 0.72 to 0.83, indicating new HFB formations. In the present study, HFB levels were not analyzed. Despite the higher FB reductions observed, the potentially newly formed HFBs, and those that resisted the baking, may pose a food safety risk since they can be further hydrolyzed to FBs during gastrointestinal digestion processes.^35^

Moreover, Meca et al.^21^ baked small maize doughs (3 g) at five different times (3 to 20 min) at different temperatures (160, 180, and 200 °C). Reductions of 53% (160 °C), 63% (180 °C), and 92% (200 °C) were reported. Under the same conditions, N-(carboxymethyl)­FB_1_ levels increased, and a BRP formed during the baking process. Even at 200 °C, the final concentrations of both N-(carboxymethyl)­FB_1_ and FB_1_ were nearly equivalent (88 and 80 μg/kg, respectively). This suggests that the thermal degradation of FBs does not necessarily lead to their destruction but rather results in the formation of different BRPs. BRPs are less hazardous than FBs due to the loss of the capability to inhibit the ceramide synthases of the animal cells.^8,21,36^ This is a result of the loss of the C2 primary amino group of FBs during the formation of BRPs. This group is essential for the mycotoxin to bind to the enzyme active site, inhibit its activity, and exert its cytotoxic effect.^21,37^

Finally, Numanoglu et al.^26^ baked traditional Turkish maize bread (1500 g) at 210 °C for 60 min and did not observe significant changes in FBs concentration.

Aforementioned studies suggest that bread in bigger sizes could affect the heat transfer, resulting in less FB_1_ degradation. For instance, Numanoglu et al.^26^ observed negligible FB reductions in larger bread sizes (1500 g). In contrast, the present study and that by Bryla et al.^22^ with smaller bread sizes (100 g) observed higher reductions. Furthermore, Meca et al.,^21^ using even smaller bread sizes (3 g), observed even higher reductions (all results previously discussed).

FBs showed reduction rates higher than those of DON during bread baking. Gbashi et al.^38^ tested the thermostability of 15 different mycotoxins in maize flour at different temperatures (103.4 to 216.6 °C) and times (6.7 to 55 min). FB_1_, FB_2_, and FB_3_ were the least thermoresistant among the rest, with average reductions of 78.3% ± 2.5%, 77.3% ± 1.8%, and 80.9% ± 2.1%, respectively. The other mycotoxins tested showed reductions ranging from 33.3% ± 3.2% to 51.0% ± 9.1%. They suggested that the more spread-out a mycotoxin molecular configuration has, the more thermolabile it tends to be, as they observed that FBs, having less compact structure than ochratoxin A or T-2 toxin, showed the highest reduction rates. This supports the idea that DON, with a small, cyclic and compact structure, presents lower thermal reduction than FBs, which have a larger, linear, and more spread-out configuration.

As observed in the case of DON, the analysis of variance indicates that the baking time plays a greater role in the reduction of FBs than the temperature applied.

### 3.3 Modeling Mycotoxin Kinetics during Bread Baking

#### 3.3.1 DON

Determination coefficients (*r* ^2^) of zero-, first-, and second-order kinetics were compared. The values obtained were very close (Table 3). Only two studies have been previously published on this issue by Vidal et al.^29^ and Numanoglu et al.^14^ on wheat and maize bread, respectively. Both determined or assumed that DON degradation followed first-order kinetics. In the present study, due to the acceptable *r* ^2^ obtained and the previous publications, first-order kinetics was selected, too.

**Table 3:** Determination of the Degradation Kinetic Order for DON^a^

	160 °C	180 °C	200 °C	220 °C
zero order (*r* ^2^)	0.701	0.922	0.894	0.928
first order (*r* ^2^)	0.720	0.937	0.877	0.920
second order (*r* ^2^)	0.738	0.948	0.852	0.901

a *r* ^2^, determination coefficient.

With the kinetic order established, the first-order eq 2 was used to estimate the kinetic parameters (Table 4).

**Table 4:** Kinetic Parameters for the Degradation of DON during Baking^a^

*T* (°C)	*k* (min^–1^)	*t*_1/2_ (min)	*r* ^2^	*E*_a_ (kJ/mol)	*r* ^2^ of *E*_a_
160	0.0016	433.1	0.720	48.9	0.997
180	0.0028	247.6	0.937		
200	0.0047	147.5	0.877		
220	0.0085	81.5	0.920		

a *T*, temperature; *k*, degradation rate constant; *t*_1/2_, compound half-life; *r* ^2^, determination coefficient; *E*_a_, energy of activation.

Degradation rate constant (*k*) doubles with each temperature increase; however, the values are very small, with the highest *k* reaching only 0.0085 min^–1^ at 220 °C. Also, half-life values (*t*_1/2_) suggest that the times required to reach half of the initial concentration are not viable in practice, as they range from 7.2 h for the lowest temperature to 1.4 h for the highest one.

From the *k* values, the activation energy (*E*_a_) was estimated. Vidal et al.^29^ and Numanoglu et al.^14^ obtained similar *E*_a_ values in their studies, reporting 46.3 and 42.8 kJ/mol, respectively, suggesting a consistent minimum energy threshold for initiating DON reduction. On the other hand, *k* values reported by those authors are much higher than those reported in this study. Numanoglu et al.^14^ observed *k* values ranging from 0.003 to 0.025 min^–1^ at temperatures of 150–250 °C, while Vidal et al.^29^ reported *k* values between 0.009 and 0.057 min^–1^ for temperatures of 140–200 °C. This difference can be due to the size difference between loaves of the present study (100 g) and those from the aforementioned authors (3 and 20 g). In this study, the sizes of the loaves are more similar to existing individual rolls or small loaves commonly produced in the industry and bakeries and closer in weight to larger bread types, such as baguettes (around 200 g). Thus, the obtained *k* values, although they are lower than those previously reported by other authors, are closer to reality.

#### 3.3.2 DON-3-Glu

In this case, due to the significant release of DON-3-Glu during the baking process, a liberation model was needed. From the several existing release kinetic models, in the present study, a first-order model was chosen. This model can be applied on water-soluble compounds in porous matrices.^39^ Since DON is a water-soluble compound and bread is a porous food matrix, the following model was used:

$$\frac{C}{C_{\max}}=1-(e^{-k_{L}\cdot t})$$ 6

which can be linearized to

$$\ln(C_{\max}-C)=\ln(C_{\max})-k_{L}\cdot t$$ 7

where *C*_max_ is the maximum compound concentration (μg/kg), *C* is the compound concentration at a certain time (μg/kg), *k*_L_ is the liberation rate constant (min^–1^), and *t* is the process time (min).

As observed in Section 3.2.1, DON-3-Glu reaches its peak release at 200 and 220 °C at 20 and 25 min, respectively, and then decreases. Therefore, eq 7 was only applied at these temperatures for the first 20 and 25 min of baking. Beyond those times, eq 2 was applied to calculate the kinetic parameters on degradation. Table 5 presents the estimated kinetic parameters.

**Table 5:** Estimated Kinetic Parameters for DON-3-Glu Release and Degradation^a^

*T* (°C)	*k*_L_ (min^–1^)	*k* (min^–1^)	*t*_2_ (min)	*t*_1/2_ (min)	release *E*_a_ (kJ/mol)	*r* ^2^
160	0.035		9.6		45.5	0.69
180	0.044		9.5			
200	0.193	0.015	1.6	44.4		
220	0.121	0.053	4.1	44.2		

a *T*, temperature; *k*, degradation rate constant; *k*_L_, liberation rate constant; *t*_2_, time to double the initial concentration; *t*_1/2_, compound half-life; *E*_a_, energy of activation; *r* ^2^, determination coefficient.

The release rate (*k*_L_) increases with the temperature, indicating faster and earlier DON-3-Glu liberation at higher temperatures compared to lower ones; consequently, the time required to double the initial concentration is short, especially at higher temperatures. Those results are aligned with the observed 460–740% DON-3-Glu increase during the initial stages of baking. Interestingly, comparing *k* and *k*_L_ estimates, it can be concluded that DON-3-Glu reduction does not occur as easily as the liberation.

#### 3.3.3 FB_1_ and FB_2_

As for DON, before estimating the kinetic parameters for FBs degradation, *r* ^2^ values were compared for the different kinetic orders (Table 6). The first- and zero-order models fitted similarly to the data, while the second- order model showed a poorer fit. Only two papers have been published on the degradation kinetics of FBs, which agrees that FB_1_ follows first-order degradation kinetics.^40,41^ Those articles focused on corn grains instead of bread, and the temperatures ranged from 50 to 150 °C, lower than the ones used in the present experiment. For these reasons, kinetic parameters were calculated for both kinetic orders, zero and first, to assess which one is more suitable.

**Table 6:** Determination of the Degradation Kinetic Order for FB_1_ and FB_2_ ^a^

		160 °C	180 °C	200 °C	220 °C
FB_1_	zero order (*r* ^2^)	0.963	0.989	0.980	0.924
	first order (*r* ^2^)	0.931	0.955	0.940	0.831
	second order (*r* ^2^)	0.886	0.979	0.859	0.718
FB_2_	zero order (*r* ^2^)	0.903	0.868	0.789	0.948
	first order (*r* ^2^)	0.930	0.886	0.811	0.910
	second order (*r* ^2^)	0.940	0.890	0.883	0.831

a *r* ^2^, determination coefficient.

Table 7 shows the kinetic parameters obtained for both studied orders. In both cases, *r* ^2^, half-lives, and energies of activation were similar.

**Table 7:** Kinetic Degradation Parameters for FBs during Baking^a^

		*T* (°C)	*k* (min^–1^)	*t*_1/2_ (min)	*E*_a_ (kJ/mol)	*r* ^2^ of *E*_a_
zero order	FB_1_	160	2.7	108.2	41.0	0.966
		180	5.8	62.3		
		200	8.3	45.3		
		220	11.2	34.1		
	FB_2_	160	0.3	150.6	44.3	0.934
		180	0.8	76.1		
		200	1.1	59.6		
		220	1.5	40.1		
first order	FB_1_	160	0.006	112.6	39.9	0.980
		180	0.012	58.7		
		200	0.019	38.2		
		220	0.020	28.9		
	FB_2_	160	0.004	176.7	42.9	0.952
		180	0.009	75.7		
		200	0.012	57.2		
		220	0.020	39.2		

a *T*, temperature; *k*, degradation rate constant; *t*_1/2_, compound half-life; *E*_a_, energy of activation.

FBs, being less thermostable than DON, require a lower *E*_a_ to initiate their degradation. Additionally, the first-order model provided the best *r* ^2^ fit for *E*_a_. According to this model, FB_1_ can be more easily reduced than FB_2_ due to its lower *E*_a_ value (39.9 kJ/mol for FB_1_ < 42.9 kJ/mol for FB_2_).

### 3.4 Mycotoxin Levels in Flour and Bread Compared to EU Permitted Maximum Levels

The European Commission has stipulated limits for the presence of several mycotoxins in flour and bakery products. DON levels are regulated by EU 2024/1022,^42^ and the summatory of FB_1_ and FB_2_ is regulated by EU 2023/915.^43^ Meanwhile, no limits are stipulated for DON-3-Glu. DON is limited to 1000 μg/kg in maize flour, to 600 μg/kg in wheat flour, and to 400 μg/kg in bread. FBs cannot exceed 2000 μg/kg in maize flour and 1000 μg/kg in maize-based food intended for direct consumption (as bread), while there is currently no limit in wheat products.

To compare the obtained results with the legislation, all toxin concentrations were recalculated on a wet basis (w.b.). If bread was produced with wheat (60%) and maize (40%) flour contaminated at the legal limits, initial concentrations of 760 μg/DON kg and 800 μg/FBs kg would be found in the flour mixture. Consequently, reductions of 48% for DON would be required to reach safe levels on the baked product according to the Regulation. In contrast, FBs would not need any reduction in this particular case; however, legislation expects a reduction of 50% from maize flour to maize bread. Table 8 shows how DON can be lowered just above the legal threshold but still at a nonaccepted level. On the other hand, bread made with FB-contaminated flour can reach safe levels after baking. DON levels increased up to 40 min of baking, whereas FB levels decreased throughout the baking process. This difference may occur due to the lower thermal stability of FBs compared to that of DON.

**Table 8:** Mycotoxin Reduction from Flour to Bread (w.b.)

	temperature (°C)	time (min)	flour (w.b.)(μg/kg)	final concentration in bread (w.b.) (μg/kg)	reduction (%)
DON	160	0	760	398.45	–47.6
		40		459.84	–39.5
		60		498.27	–34.4
	180	0		413.98	–45.5
		40		473.56	–37.7
		60		481.97	–36.6
	200	0		395.07	–48.0
		40		465.09	–38.8
		60		429.83	–43.4
	220	0		410.32	–46.0
		20		441.91	–41.9
		40		405.36	–46.7
FBs	160	0	800	254.35	–68.2
		40		268.25	–66.5
		60		285.13	–64.4
	180	0		316.05	–60.5
		40		284.29	–64.5
		60		236.72	–70.4
	200	0		302.16	–62.2
		40		287.73	–64.0
		60		178.00	–77.7
	220	0		308.20	–61.5
		20		281.17	–64.9
		40		170.85	–78.6

In conclusion, DON and FBs concentrations in bread were significantly reduced under all baking conditions tested. Specifically, the baking time had more impact than the temperature applied. DON showed more thermal stability than FBs under all conditions, probably due to their different structural configuration. FB_1_ was more affected by temperature than FB_2_. The DON-3-Glu concentration showed notable increases during the early stages of baking; after that, when lower temperatures were applied, it remained stable throughout the process; however, with higher temperatures, it was reduced to the initial concentration. Using a first-order reaction model, *E*_a_ was calculated for DON (48.9 kJ/mol), which was similar to the existing data in the literature. *E*_a_ was calculated for thermal degradation of FBs in bread for the first time (39.9 and 42.9 kJ/mol for FB_1_ and FB_2_, respectively). Regarding DON-3-Glu, liberation kinetics have been applied. During the initial stages of baking, the *E*_a_ needed for its release was 45.5 kJ/mol. The reported kinetic parameters should be valuable tools for the food industry to produce safer food products and optimize their thermal processes to offer products organoleptically acceptable and safe while being more energetically sustainable. Additionally, new baking conditions can be developed to lower certain initial mycotoxin levels to meet the current legal limits. This is particularly critical for DON, as baking processes may not be enough to reduce its levels to safe limits in bread made from flour containing DON levels below the legal threshold.

## Abbreviations

DON: deoxynivalenol
DON-3-Glu: deoxynivalenol-3-β-d-glucoside
FBs: B-type fumonisins
FB_1_: fumonisin B_1_
FB_2_: fumonisin B_2_
HPLC: high-performance liquid chromatography
BRP: browning reaction product
